# A genomic data archive from the Network for Pancreatic Organ donors with Diabetes

**DOI:** 10.1038/s41597-023-02244-6

**Published:** 2023-05-26

**Authors:** Daniel J. Perry, Melanie R. Shapiro, Sonya W. Chamberlain, Irina Kusmartseva, Srikar Chamala, Leandro Balzano-Nogueira, Mingder Yang, Jason O. Brant, Maigan Brusko, MacKenzie D. Williams, Kieran M. McGrail, James McNichols, Leeana D. Peters, Amanda L. Posgai, John S. Kaddis, Clayton E. Mathews, Clive H. Wasserfall, Bobbie-Jo M. Webb-Robertson, Martha Campbell-Thompson, Desmond Schatz, Carmella Evans-Molina, Alberto Pugliese, Patrick Concannon, Mark S. Anderson, Michael S. German, Chester E. Chamberlain, Mark A. Atkinson, Todd M. Brusko

**Affiliations:** 1 Department of Pathology, Immunology and Laboratory Medicine, Diabetes Institute, College of Medicine, University of Florida, Gainesville, FL 32611 USA; 2 Diabetes Center, School of Medicine, University of California San Francisco, San Francisco, CA 94143 USA; 3 Department of Biostatistics, College of Public Health and Health Professions, University of Florida, Gainesville, FL 32610 USA; 4 Department of Diabetes and Cancer Discovery Science, Arthur Riggs Diabetes and Metabolism Research Institute, Beckman Research Institute, City of Hope, Duarte, CA 91010 USA; 5 Department of Pediatrics, Diabetes Institute, College of Medicine, University of Florida, Gainesville, FL 32610 USA; 6 Biological Sciences Division, Earth and Biological Sciences Directorate, Pacific Northwest National Laboratory, Richland, WA 99352 USA; 7 Department of Biomedical Engineering, College of Engineering, University of Florida, Gainesville, FL 32611 USA; 8 Center for Diabetes and Metabolic Diseases and the Wells Center for Pediatric Research, Indiana University School of Medicine, Indianapolis, IN 46202 USA; 9 Diabetes Research Institute, Department of Medicine, Division of Endocrinology, Diabetes and Metabolism, Department of Microbiology and Immunology, Miller School of Medicine, University of Miami, Miami, FL 33021 USA; 10 Genetics Institute, University of Florida, Gainesville, FL 32601 USA

**Keywords:** Type 1 diabetes, Disease genetics, Diabetes

## Abstract

The Network for Pancreatic Organ donors with Diabetes (nPOD) is the largest biorepository of human pancreata and associated immune organs from donors with type 1 diabetes (T1D), maturity-onset diabetes of the young (MODY), cystic fibrosis-related diabetes (CFRD), type 2 diabetes (T2D), gestational diabetes, islet autoantibody positivity (AAb+), and without diabetes. nPOD recovers, processes, analyzes, and distributes high-quality biospecimens, collected using optimized standard operating procedures, and associated de-identified data/metadata to researchers around the world. Herein describes the release of high-parameter genotyping data from this collection. 372 donors were genotyped using a custom precision medicine single nucleotide polymorphism (SNP) microarray. Data were technically validated using published algorithms to evaluate donor relatedness, ancestry, imputed HLA, and T1D genetic risk score. Additionally, 207 donors were assessed for rare known and novel coding region variants via whole exome sequencing (WES). These data are publicly-available to enable genotype-specific sample requests and the study of novel genotype:phenotype associations, aiding in the mission of nPOD to enhance understanding of diabetes pathogenesis to promote the development of novel therapies.

## Background & Summary

**Fig. 1:**
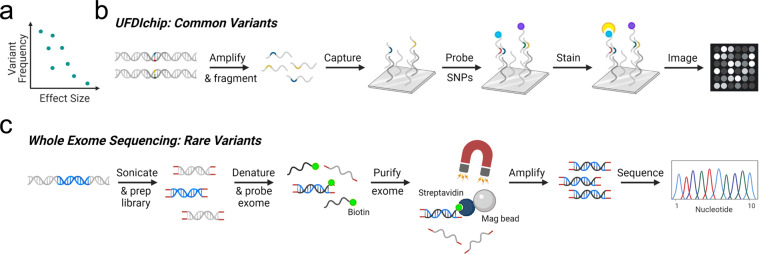
Complementary methods for detecting genetic variants associated with T1D. (**a**) The effect size of any given variant on T1D risk is inversely related to the frequency of the variant^5^. (**b**) To detect more commonly observed variants, DNA samples from nPOD donors were probed for the presence of SNPs previously reported in T1D GWAS^2^ efforts using the UFDIchip, yielding CEL microarray image files (modified from Affymetrix Axiom website: https://www.affymetrix.com/products_services/arrays/specific/axiom_mydesign.affx). (**c**) To detect rare or novel variants, whole exome sequencing (WES) was performed on DNA from nPOD donors (modified from Roche NimbleGen SeqCap EZ Exome Library workflow^68^). Diagrams created in BioRender.

Genetic predisposition to risk for or protection from type 1 diabetes (T1D) is highly polygenic, with the total possible set of disease-associated variants yet to be fully defined^1^. Genome-wide association studies (GWAS) have identified population-level risk loci (minor allele frequency (MAF) >1%), dominated by Human Leukocyte Antigen (HLA) class II and insulin, and accompanied by 77 additional regions, which in total cover over 3,600 predicted causal moderate effect size variants (odds ratio (OR) <2) associated with genes thought to impact leukocyte and pancreatic β-cell function^2,3^. While a combination of many such population-level variants may contribute to the development of “classical” T1D^2,3^ and latent autoimmune diabetes in adults (LADA)^4^, we are beginning to appreciate that rare (MAF ≤1%) larger effect size (OR ≥2) variants may explain the “missing heritability” of autoimmune diabetes^5^ (Fig. 1a). In support of this notion, rare variants with large effect size are associated with monogenic autoimmune forms of diabetes including immune dysregulation, polyendocrinopathy, enteropathy, X-linked syndrome (IPEX), signal transducer and activator of transcription 3 (*STAT3*)-, and cytotoxic T-lymphocyte protein 4 (*CTLA4*)-associated diabetes^6^, in addition to non-autoimmune forms of diabetes such as maturity-onset diabetes of the young (MODY) and cystic fibrosis-related diabetes (CFRD)^7^.

The Network for Pancreatic Organ donors with Diabetes (nPOD)^8^, founded in 2007, has become the largest biorepository of human pancreata, pancreatic lymph nodes, and spleen from organ donors with T1D, MODY, CFRD, T2D, gestational diabetes, non-diabetic islet autoantibody positive (AAb+) donors, and non-diabetic autoantibody-negative (AAb-) control donors^9^. nPOD provides worldwide distribution of biospecimens to researchers working to elucidate T1D pathogenesis in order to promote the development of new strategies for prevention and treatment. To date (September 2022), nPOD has supplied biosamples to > 280 independent research projects studying β-cell physiology, β-cell differentiation, immunology, T1D biomarkers, technology development, T1D pathology, and diabetes etiology (https://www.jdrfnpod.org/publications/current-npod-projects/, accessed October 21, 2021). A major goal of nPOD, in addition to biosample distribution, is the sharing of de-identified donor data from multiple core laboratories to facilitate discovery efforts. The nPOD Data Portal provides approved investigators with access to donor clinical and demographic information, serum HbA1c and C-peptide levels, islet AAb status (insulin, glutamic acid decarboxylase [GAD], insulinoma-associated antigen-2 [IA-2], zinc transporter 8 [ZnT8])^10^, pancreas weights, and histopathology reviews^8,11^ (https://portal.jdrfnpod.org/, accessed October 21, 2022). Whole slide scans from hematoxylin and eosin-stained (H&E) sections are available for online viewing via the Online Pathology portal (https://aperioeslide.ahc.ufl.edu/, accessed October 21, 2022) for access to cross-sectional pancreas morphology as well as multiplex immunohistochemistry (IHC)-stained sections for insulin, glucagon, somatostatin, and pancreatic polypeptide (PP) to visualize endocrine β-, α-, δ-, and PP cells, respectively. Multiplex IHC staining panels are also available for markers including, but not limited to, Ki67, CD3, insulin, and/or glucagon for quantification of cell proliferation and immune cell infiltration in pancreatic endocrine and exocrine compartments. Histopathology reports summarizing blinded assessment of H&E- and IHC-stained sections from pancreas and other available organs are provided to detail islet parameters and major abnormalities. In terms of genetic data, the standard operating procedures (SOPs) for nPOD donors were previously limited to the collection of high-resolution four-digit HLA genotypes^8,12^. The availability of additional high-parameter genotyping data has therefore been a high priority that is now realized with the data release described herein.

Our approach for characterizing nPOD donor genetics was twofold: donors were genotyped with 1) the University of Florida Diabetes Institute (UFDI) custom single nucleotide polymorphism (SNP) microarray (UFDIchip)^13^ and 2) the University of California San Francisco (UCSF) standardized whole exome sequencing (WES) pipeline^14^. Specifically, nPOD cases (N = 372)— comprised of AAb- no diabetes controls (N = 147), AAb + without T1D (N = 26), T1D (N = 111), T1D medalists^15,16^ (N = 2), T1D recipients of pancreas transplant (N = 5), type 2 diabetes (T2D, N = 38), gastric bypass (N = 2), gestational diabetes (N = 4), monogenic diabetes (N = 4), cystic fibrosis (CF, N = 5), other diabetes (N = 12), other no diabetes (N = 12), and pregnant without diabetes (N = 4)— were genotyped using the UFDIchip^13^ custom Axiom^TM^ array (Fig. 1b). All nPOD donors with available DNA or tissue were evaluated for population-level variants via UFDIchip. We prioritized the selection of T1D, AAb + without T1D, gestational diabetes, monogenic diabetes, and other diabetes donors in addition to including a few no diabetes donors as controls for WES-based characterization of rare diabetes-associated variants that may not have been powered for detection by previous GWAS studies. Specifically, nPOD donors (N = 207)— including AAb- no diabetes controls (N = 13), AAb + without T1D (N = 34), T1D (N = 135), T1D recipients of pancreas transplant (N = 6), gestational diabetes (N = 4), monogenic diabetes (N = 4), and other diabetes (N = 11)— were queried for rare known and novel coding region variants in autoimmune and MODY-associated genes via WES^14^ (Fig. 1c). Data emanating from these assays were used to provide individual genotypes, infer relatedness^17^ and genetic ancestry^18^, impute HLA^19^, and calculate a combined T1D genetic risk score (GRS)^20–22^ per donor.

These genotyping data have been generated and made accessible to enable genotype-selected sample requests and the study of novel genotype:phenotype associations by the international community of nPOD investigators. We anticipate that the diversity of nPOD donor genetics may be partly responsible for inter-donor heterogeneity observed in islet health, insulitis composition, age at T1D onset, islet AAb status, and other endotype-related characteristics^23–25^. Importantly, beyond explaining diabetes heterogeneity, the findings facilitated by these data are expected to inform precision medicine strategies for prevention or suspension of the pathogenesis of T1D as well as other forms of diabetes.

## Methods

### Donor tissues

Transplant-quality organs, including pancreas and up to 13 other tissues, were recovered from cadaveric organ donors by United States (U.S.) organ procurement organizations (OPOs, http://www.jdrfnpod.org//for-partners/npod-partners/, accessed October 15, 2021) in accordance with federal guidelines, then processed by the nPOD Organ Processing and Pathology Core (OPPC) according to University of Florida (UF) Institutional Review Board (IRB) approved protocol IRB201600029, as previously described^8,11^. Studies conducted using organ donor tissue samples from the nPOD biobank are classified as minimum risk research, as study participants are no longer living. However, informed consent for research participation is obtained from family members via both written and verbal communication prior to organ donation, with the consent processes undertaken by qualified personnel affiliated with the U.S. OPO network. All subject information is de-identified in accordance with HIPAA regulations. For each donor, clinical and demographic information, were obtained via medical chart review and OPO-conducted interview with the donor’s family. High-resolution four-digit HLA typing was performed by Next Generation Sequencing (NGS) as previously described^8,12^ at the Barbara Davis Center for Childhood Diabetes HLA Core (University of Colorado Anschutz Medical Campus). nPOD donors were categorized by diabetes type, verified by UF endocrinologist review of the de-identified terminal medical records (including diagnosis and duration of diabetes, history or clinical data for diabetic ketoacidosis, medications, and BMI), donor metadata (e.g., age, sex, reported race and ethnicity), and additional data (serum C-peptide levels, islet AAb status^10^, hemoglobin A1c [HbA1c], and high-resolution HLA^8,12^). Unique research resource identifiers (RRIDs) were assigned to each organ donor, in order to facilitate the provenance and reproducibility of results^26^.

### DNA isolation

DNA was extracted from frozen spleen or, for a limited number of cases in which spleen was unavailable, frozen pancreas, pancreatic lymph node, or small intestine were used. DNA isolation was performed using the Qiagen DNeasy Blood and Tissue DNA isolation kit according to the manufacturer’s instructions. Purity and concentration of extracted DNA were assessed with the Epoch Microplate Spectrophotometer (BioTek).

### UFDIchip design

**Fig. 2:**
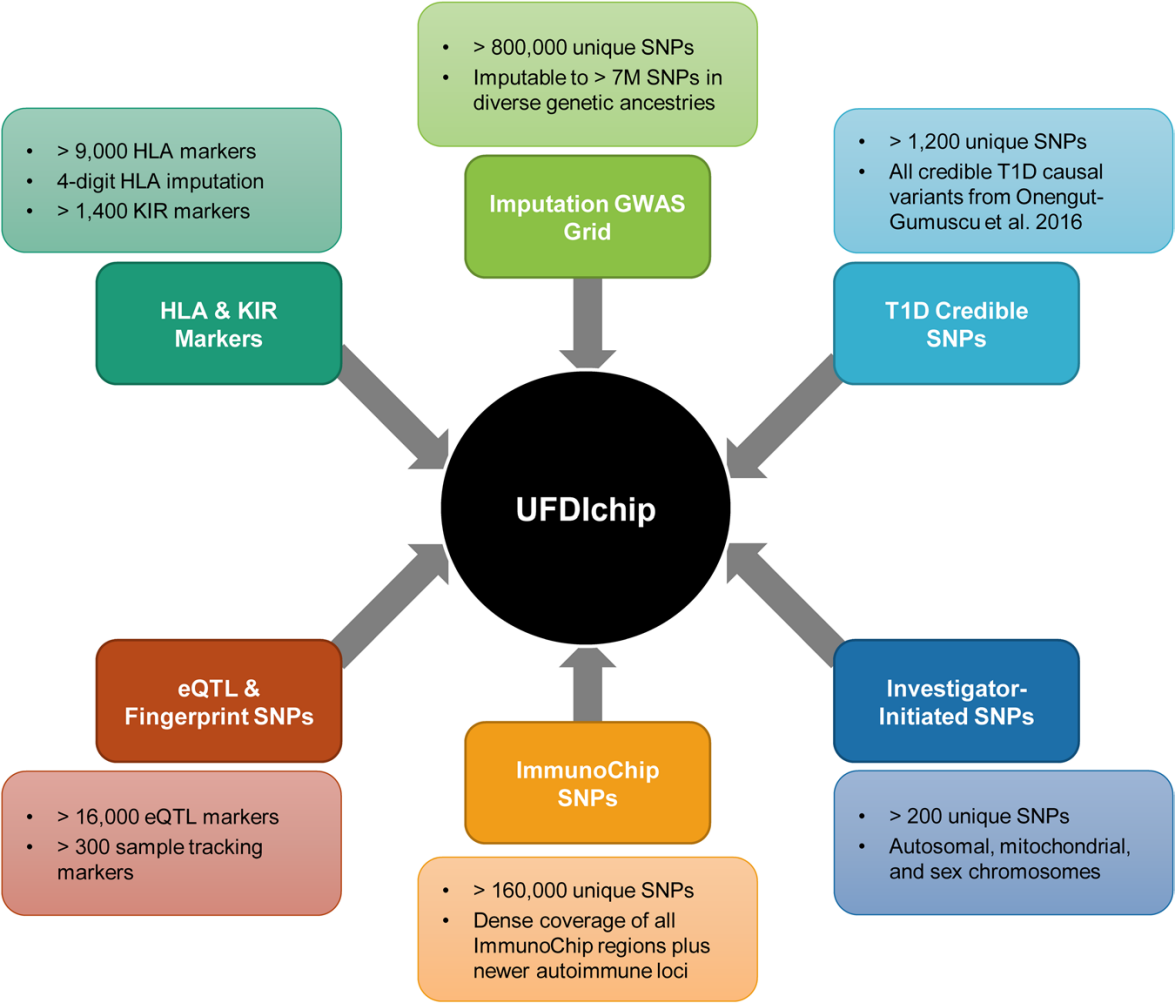
University of Florida Diabetes Institute (UFDI) chip design. The UFDIchip contains probes from the following modules of the Axiom^TM^ Precision Medicine Research Array (PMRA, Thermo Fisher Scientific): imputation genome-wide association study (GWAS) grid, human leukocyte antigens (HLA), killer cell immunoglobulin-like receptors (KIR), expression quantitative trait loci (eQTL), and fingerprinting/sample tracking. Custom additions to the UFDchip included single nucleotide polymorphisms (SNPs) from the following categories: ImmunoChip v2, credible type 1 diabetes (T1D) causal variants^3^, and investigator-initiated markers of interest.

372 nPOD donors (Table 1, **Phenotype_data**.**txt**^27^) were genotyped at 985,971 unique loci on a custom SNP array termed the UFDIchip^13^ (Fig. 1b). The base array is the Axiom^TM^ Precision Medicine Research Array (Thermo Fisher Scientific), to which all content from the ImmunoChip v2^28^ was added, as well as all previously reported credible T1D risk variants^3^ (Fig. 2, **UFDIchip_library_file**.**xlsx**^27^). The array also includes dense coverage of the highly polymorphic HLA region, which allows for accurate imputation of HLA haplotypes to 4-digit resolution.

**Table 1:** nPOD donors genotyped by UFDIchip.

Clinical Group	Female	Male	Black/African American	American Indian/Alaska Native	Asian	White/Caucasian	Hispanic/Latinx	Multiracial	Age (Median [IQR])	Diabetes Duration (Median [IQR])
No Diabetes	50	97	30	1	1	93	20	2	15.20 [3.9–25.9]	N/A
AAb +	12	14	2	0	0	18	6	0	25.17 [21.0–38.5]	N/A
T1D	52	59	16	0	0	86	9	0	26.00 [19.1–33.9]	11.00 [5.0–20.0]
T1D Medalist	1	1	0	0	0	2	0	0	69.00 [64.5–73.5]	66.50 [62.8–70.2]
Transplant	1	4	0	0	0	5	0	0	52.00 [47.0–55.3]	38.00 [26.0–40.0]
T2D	18	20	11	0	2	17	8	0	48.90 [45.0–60.0]	10.00 [3.0–15.0]
Gastric Bypass	2	0	0	0	0	2	0	0	42.00 [39.0–45.0]	3.00 [3.0–3.0]
Gestational Diabetes	4	0	1	0	0	2	1	0	33.35 [32.2–33.9]	0.06 [0.1–0.1]
Monogenic Diabetes	2	2	1	0	0	2	1	0	33.55 [24.6–41.7]	17.50 [12.0–22.0]
Cystic Fibrosis	3	2	0	0	0	4	1	0	31.10 [29.3–33.0]	5.00 [2.0–7.0]
Other-Diabetes	6	6	2	0	0	7	3	0	33.95 [26.0–44.5]	4.00 [2.0–13.5]
Other-No Diabetes	6	6	0	0	1	8	3	0	17.52 [14.8–23.0]	N/A
Pregnancy	4	0	2	1	1	0	0	0	29.45 [21.4–37.0]	N/A
Total	161	211	65	2	5	246	52	2		

Data are presented as number of male and female donors and number of donors per reported race/ethnicity for each clinical group, along with age (years) and diabetes duration (years). AAb+ : islet autoantibody positive without T1D, T1D: Type 1 diabetes, T1D Medalist: T1D duration > 50 years^15,16^, Transplant: Medical history of T1D and pancreas transplant recipient, T2D: Type 2 diabetes, N/A: not applicable, IQR: interquartile range.

### Genotype processing and analysis

UFDIchip plates were processed on an Affymetrix GeneTitan instrument with external sample handling on a BioMek FX dual arm robotic workstation. Axiom™ Analysis Suite software (v3.0, Thermo Fisher Scientific) was used to process raw CEL file data to plink text files. The software includes quality control (QC) procedures at the sample, plate, and SNP levels. These QC threshold parameters were set to Axiom™ Analysis Suite default stringency (“Best Practices Workflow” using “Human.legacy.v5” settings). Under these settings, samples were included in analysis if dish QC (DQC) ≥ 0.82 and if QC call rate ≥ 97%. Plates were considered acceptable for analysis if average QC call rate ≥ 98.5% for passing samples. Best probe set was identified per SNP, with the SNP call rate threshold set to 95%. A screen for discordance from reported sex via X chromosome heterozygosity was then performed using plink v1.9^29^. All data passed these QC screens and raw CEL files and a binary plink file containing processed data (GRCh37/hg19) from all cases are stored in the database of Genotypes and Phenotypes (dbGaP)^27^. Subsequent analyses included relatedness estimation using KING^30^, genetic ancestry imputation using ADMIXTURE^18^, HLA imputation using Axiom^TM^ HLA Analysis Software^19^, imputation to 300 M SNPs and indels using the Trans-Omics for Precision Medicine (TOPMed) reference cohort with the Michigan Imputation Server^31^, and calculation of a T1D GRS^21,22^.

### Validation of technical replicates

DNA from 24 nPOD donors were run in duplicate on the UFDIchip. SNP call rates were compared between technical replicates using Bland-Altman analysis. Reproducibility of genotype calls between technical replicates were evaluated by kinship coefficient using KING^30^ (v2.1.2) software.

### Relatedness

Genotyping data from the nPOD cohort with unknown and from the 1000 Genomes phase 3 cohort^32^ with known family relationships were merged and analyzed for genetic relatedness using KING^30^ (v2.1.2) software. The integrated relationship inference command was used to infer up to third-degree relatives. Relationships between nPOD case pairs and between 1000 Genomes pairs were represented by plotting estimated kinship coefficients. Kinship coefficients of unrelated 1000 Genomes pairs were randomly downsampled to the number of nPOD subject pairs to allow for data visualization.

### Genetic ancestry

Data from unrelated subjects from the 1000 Genomes phase 3 cohort^32^ were filtered for SNPs that overlap with the UFDIchip array using plink v1.9^29^. The data were pruned for linkage disequilibrium (LD) by removing SNPs with R^2^ > 0.1, screening within a 50 SNP block and proceeding by steps of 10 SNPs. This yielded 1000 Genomes genotypes for 320,005 SNPs, which were used to run an unsupervised analysis using ADMIXTURE software^18^ (v1.3.0) with k set to five populations. Each of the five groups represented a unique continental population from 1000 Genomes and as such, were assigned: 1) African (AFR), 2) Admixed American (AMR), 3) East Asian (EAS), 4) European (EUR), and 5) South Asian (SAS)^32^. The 372 nPOD cases were then projected onto the reference population to estimate ancestry proportions. Dimensionality reduction of the resulting Q-values (ancestry proportions) was performed using principal component analysis (PCA) to enable visualization.

### HLA Imputation

Axiom^TM^ HLA Analysis Software (v1.2.0.38)^19^ was used to impute 2-digit and 4-digit HLA genotypes, along with probability scores for the imputed calls. Concordance with nPOD HLA typing results^8^ was assessed at *HLA-A*, *HLA-DRB1*, *HLA-DQA1*, and *HLA-DQB1*. The typed result was considered ground truth when the imputed result was discordant.

Imputation accuracy for each of these loci [*Acc(L)*] was calculated as previously reported^33^, substituting the dosage for the probability score that is provided by Axiom^TM^ HLA Analysis Software^19^:

$$Acc\left(L\right)=\frac{{\sum }_{i=1}^{n}{P}_{i}\left(A{1}_{i,L}\right)+{P}_{i}\left(A{2}_{i,L}\right)}{2n}$$

where *P*_*i*_ is the probability for imputed alleles *A1*_*i,L*_ and *A2*_*i,L*_ for donor *i* at locus *L*. Imputed alleles were considered concordant when they were included in the donor’s set of typed alleles at locus *L*, and discordant when they were not in the set of typed alleles at locus *L*. For discordant alleles, *P*_*i*_ was set to 0. The summation of probabilities for the total number of donors assessed, *n*, was then divided by the total number of alleles tested, 2*n*. The accuracy score ranges from 0, for no concordant calls, to 1, for complete concordance with probabilities of 1 for all alleles.

Concordance was calculated at the 2-digit and 4-digit level for genotypes related to T1D risk or protection, as determined in primarily White cohorts^34–36^. These included HLA-A*02:01, HLA-A*24:02, HLA-DRB1*03:01 (DR3), HLA-DQA1*05:01–HLA-DQB1*02:01 (DQ2), HLA-DRB1*04:xx (DR4), HLA-DQA1*03:01–HLA-DQB1*03:02 (DQ8), HLA-DRB1*08:01 (DR8), HLA-DQA1*04:01–HLA-DQB1*04:02 (DQ4), HLA-DRB1*15:xx (DR15), HLA-DQA1*01:02–HLA-DQB1*06:02 (DQ6), and HLA-DQA1*03:01–HLA-DQB1*03:01 (DQ7), where xx is any sub-allele. The following formula was used:

$$Concordance=\frac{{\sum }_{i=1}^{n}\,A{1}_{i,L}+A{2}_{i,L}}{{\sum }_{i=1}^{n}\,A{1}_{t,L}+A{2}_{t,L}}$$

where the number of imputed alleles *A1*_*i,L*_ and *A2*_*i,L*_ matching the genotype of interest for donor *i* at locus *L* was summed across all donors, *n*, and divided by the number of typed alleles *A1*_*t,L*_ and *A2*_*t,L*_ matching the genotype of interest for donor *i* at locus *L* summed across all donors. The accuracy score ranges from 0, for no concordant calls, to 1, for complete concordance.

Donor-level imputation accuracy [*Acc(S)*] was calculated as:

$$Acc\left(S\right)=\frac{{\sum }_{j=1}^{n}{P}_{j}\left(A{1}_{j,S}\right)+{P}_{j}\left(A{2}_{j,S}\right)}{2n}$$

where *P*_*j*_ is the probability for imputed alleles *A1*_*j,S*_ and *A2*_*j,S*_ at each HLA locus *j* of donor *S*. Concordance was determined as described above, and *P*_*j*_ was set to 0 for discordant alleles. The total number of loci tested, *n*, was 4 per donor (*HLA-A*, *HLA-DRB1*, *HLA-DQA1*, and *HLA-DQB1*). The accuracy score ranges from 0, for no concordant calls, to 1, for complete concordance with probabilities of 1 for both alleles at each locus for donor *S*.

### T1D GRS calculation

We computed polygenic T1D genetic risk scores, referred to as GRS1^21,22^, GRS2^37^, and African-Ancestry (AA)-GRS^38^. GRS1 is calculated using dosages of risk genotypes for 30 T1D-associated SNPs^21^. Genotypes were obtained by imputing to the TOPMed (v r2)^31^ panel (R^2^ > 0.97). rs2187668 was not found in TOPMed, thus, a suitable proxy SNP from GRS2^37^, rs9273369, was used instead. The HLA component of GRS1 was calculated using the Polygenic Risk Score (PRS) Toolkit for HLA (v0.22a) developed by Sharp *et al*.^37^. The non-HLA component of GRS1 was then calculated via weighted sum, using odds ratios from Oram *et al*.^21^. The HLA and non-HLA scores were summed and normalized as described in Oram *et al*.^21^. GRS2 is calculated using dosages of risk genotypes for 67 T1D-associated SNPs^37^. Genotypes were obtained by imputing to the TOPMed (v r2)^31^ panel (R^2^ > 0.97). rs2476601, rs1281934, rs9273342, rs9271346, rs1233320, rs16822632, rs116522341, rs559242105, and rs371250843 were not found in TOPMed, thus, suitable proxy SNPs rs6679677, rs1281943, rs9273032, rs9271347, rs1233320, rs17840116, rs9268500, rs3129197, and rs9266268 were respectively used instead. The HLA component of GRS2 was calculated using the PRS Toolkit for HLA (v0.22a) developed by Sharp *et al*.^37^. The non-HLA component of GRS2 was then calculated via weighted sum, using odds ratios from Sharp *et al*.^37^ and added to the HLA component. AA-GRS is calculated using dosages of risk genotypes for 7 T1D-associated SNPs^38^. Genotypes were obtained by imputing to the TOPMed (v r2)^31^ panel (R^2^ > 0.96). rs2187668 and rs34303755 were not found in TOPMed; thus, suitable proxy SNPs rs9273369 and rs9268838 were respectively used instead. The AA-GRS was then calculated via weighted sum, using odds ratios from Onengut-Gumuscu *et al*.^38^.

### WES

For 207 nPOD donors (Table 2, **Phenotype_data**.**txt**^27^), WES libraries were generated as previously described^39^ (Fig. 1c) using the Agilent SureSelect Human All Exon kit (Agilent Technologies, CA, USA). Procedures and quality control (QC) measures were performed following manufacturer’s recommendations. Briefly, 180–280 bp fragments were generated from genomic DNA by sonication (Covaris) with exonuclease and polymerase subsequently utilized to convert remaining overhangs into blunt ends. The DNA fragments were adenylated on the 3′ ends followed by ligation of adapter oligonucleotides. Successfully ligated DNA fragments were enriched by PCR. Following hybridization with biotin-labelled probes, exons were captured with streptavidin-coated magnetic beads. After a wash, probes were digested. Libraries were enriched and index tags added by PCR. Amplified exon libraries were purified using AMPure XP (Beckman Coulter), quantified by Agilent high sensitivity DNA kit using an Agilent Bioanalyzer 2100, then sequenced via Illumina Novaseq. 6000 (Illumina, CA, USA). Burrows-Wheeler Aligner (BWA, v0.7.17) was utilized to map the paired-end clean reads to the GRCh37/hg19 human reference genome^40^. Genome Analysis Toolkit (GATK, v4.1.2.0) was employed for SNP/InDel detection^41^. Annotate Variation (ANNOVAR, v20191024) was used for variant annotation^42^. Other variant annotations were performed using American College of Medical Genetics (ACMG) Classification^43^, Sorting Intolerant from Tolerant (SIFT) Function Prediction (SIFT4G)^44^, PolyPhen-2 Function Prediction (v 2.2.2)^45^, Combined Annotation Dependent Depletion (CADD, v1.6) Score^46^, Genome Aggregation Database (gnomAD, v2.1.1) frequency^47^, Human Gene Mutation Database (HGMD professional 2020.2)^48^, ClinVar (accessed August 31, 2020)^49^ and Centogene Mutation Database (CentoMD, v5.8)^50^. All data passed these QC screens and are stored in dbGaP^27^.

**Table 2:** nPOD donors subjected to WES.

Clinical Group	Female	Male	Black/African American	American Indian/Alaska Native	Asian	White/Caucasian	Hispanic/Latinx	Multiracial	Age (Median [IQR])	Diabetes Duration (Median [IQR])
No Diabetes	4	9	2	1	0	8	2	0	19.00 [7.8–24.5]	N/A
AAb+	12	22	5	0	0	21	8	0	25.17 [22.0–36.4]	N/A
T1D	62	73	21	0	0	105	9	0	24.00 [17.5–32.6]	10.00 [5.0–19.0]
Transplant	0	6	0	0	0	4	2	0	42.34 [37.2–53.2]	28.00 [20.0–36.0]
Gestational Diabetes	4	0	1	0	0	2	1	0	33.35 [32.2–33.9]	0.06 [0.1–0.1]
Monogenic Diabetes	2	2	1	0	0	2	1	0	33.55 [24.6–41.7]	17.50 [12.0–22.0]
Other-Diabetes	5	6	2	0	0	7	2	0	34.00 [29.0–47.0]	3.50 [1.5–14.2]
Total	88	118	32	1	0	148	25	0		

Data are presented as number of male and female donors and number of donors per reported race/ethnicity, along with age (years) and diabetes duration (years). AAb+ : islet autoantibody positive, T1D: Type 1 diabetes, N/A: not applicable, IQR: interquartile range.

### UFDIchip and WES comparison

For 167 nPOD donors, both UFDIchip- (Table 1, **Phenotyp_data**.**txt**^27^) and WES-based (Table 2, **Phenotype_data**.**txt**^27^) genotyping were performed. Biallelic autosomal variants detected in both assays and with at least one minor allele count (MAC) in the WES data were filtered using plink v1.9^29^, resulting in 27,852 variants for comparison. Per-SNP intra-assay concordance levels were calculated across all subjects.

## Data Records

UFDIchip array data are stored in dbGaP^27^ as raw CEL files and compiled processed data from all donors deposited as binary plink files (hg19). All genotyped donors, as well as their age, sex, reported race, diabetes status and duration, are provided in **Phenotype_data**.**txt**^27^ with additional donor information available on the nPOD Data Portal (https://portal.jdrfnpod.org/, accessed October 21, 2022).

WES data are stored in dbGaP^27^, including raw exome sequencing data files (fastq format) or hg19 aligned exome sequencing data (bam format), in addition to processed variant files (vcf format). A spreadsheet listing variants and associated annotations per donor, as described in the methods, was also submitted (csv format). All donors subjected to WES, as well as their age, sex, reported race, diabetes status and duration, are listed in **Phenotype_data**.**txt**^27^ with additional donor information available on the nPOD Data Portal (https://portal.jdrfnpod.org/, accessed October 21, 2022).

## Technical Validation

### Quality control assessment of the UFDIchip genotype array

**Fig. 3:**
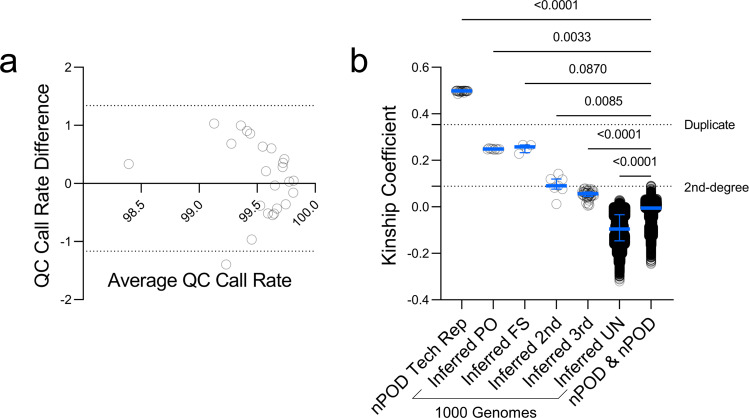
UFDIchip data are highly replicable and nPOD donors are unrelated. (**a**) Bland-Altman plot showing average vs. difference in QC call rates for n = 24 technical replicates. Horizontal dashed lines indicate 95% limits of agreement. (**b**) Relatedness analysis was performed using KING software^30^ for genotyping data from 372 nPOD donors, 24 of which were run in technical replicate, along with 2,504 1000 Genomes phase 3 cohort^32^ subjects, known to include some closely related individuals. Relatedness assessed via estimated kinship coefficients. Kinship coefficients from nPOD donor pairs were compared to those from 1000 Genomes subject pairs including inferred parent-offspring (PO), first-degree siblings (FS), second-degree relatives (2^nd^), third-degree relatives (3^rd^), and unrelated (UN). Bars represent median and interquartile range (IQR). Horizontal dashed lines indicate lower cutoffs for duplicate samples (kinship coefficient = 0.354) and second-degree relatives (kinship coefficient = 0.0884). Kruskal-Wallis test with Dunn’s multiple comparisons test for nPOD & nPOD (different subjects) versus all inferred 1000 Genomes subject relationship types or nPOD technical replicates.

As of this report, 372 nPOD donors have been genotyped on the UFDIchip and the resulting data are accessible on dbGaP (see Data Records). All array results were subjected to basic QC analyses that assessed donor-level DQC; donor-, plate-, and SNP-level call rate; and sex concordance. Donor DQC or call rate failures were re-processed with freshly extracted DNA when necessary. nPOD samples were batch-processed with data from living donors^20^ to facilitate calling of low-frequency variants^51^, resulting in 942,466 high quality genotypes passing the SNP call rate threshold. The nPOD cohort demonstrated SNP call rates of 99.58 [99.19–99.84] (median [interquartile range (IQR)]). All nPOD samples were assessed for concordance between reported and imputed sex according to level of X chromosome heterozygosity using plink v1.9^29^. For all nPOD cases, imputed sex matched reported sex. Thus, all 372 nPOD samples passed basic QC measures. Additionally, 24 nPOD samples were run in technical replicate to assess assay reproducibility. Call rates between the technical replicates differed minimally, with 0.087 ± 0.640% bias (mean ± standard deviation, Fig. 3a). Importantly, the kinship coefficients between the 24 technical replicates were 0.499 [0.496–0.499] (median [IQR]), suggesting near identical genotype calls (Fig. 3b).

### Relatedness estimation

Next, relatedness between donors was assessed. Due to the nature of donor organ procurement, it is highly improbable, although not impossible, that nPOD donors may be related. A relatedness analysis of the 372 nPOD donors (69,006 possible pair combinations) using KING software^30^ found that all of these donor pairs were inferred to be unrelated (>third-degree relatives). For comparison, we also assessed the relatedness of 2,504 1000 Genomes phase 3 cohort^32^ subjects. While this set was designed to consist of unrelated individuals, it unintentionally included a few first-, second-, and third-degree relatives^32^. When relatedness between nPOD donor pairs was compared to relatedness between 1000 Genomes^32^ subject pairs, nPOD donor pairs showed significantly smaller kinship coefficients than inferred parent-offspring (PO), full sibling (FS), 2^nd^ degree relative, and 3^rd^ degree relative pairs from 1000 Genomes (Fig. 3b), suggesting that nPOD donors are not closely related. Note that nPOD donor pairs had significantly larger kinship coefficients than inferred unrelated (UN) pairs from 1000 Genomes^32^ (Fig. 3b), potentially due to increased similarity in genetic ancestry^52^ between subjects in the nPOD cohort than in the 1000 Genomes cohort, which was specifically designed to sample individuals with diverse genetic ancestry. Beyond confirming expected relatedness in the nPOD cohort, this validates that users of this resource may employ population-level quantitative trait locus (QTL) analysis methods with these genetic data.

### Alignment with genetic ancestry

**Fig. 4:**
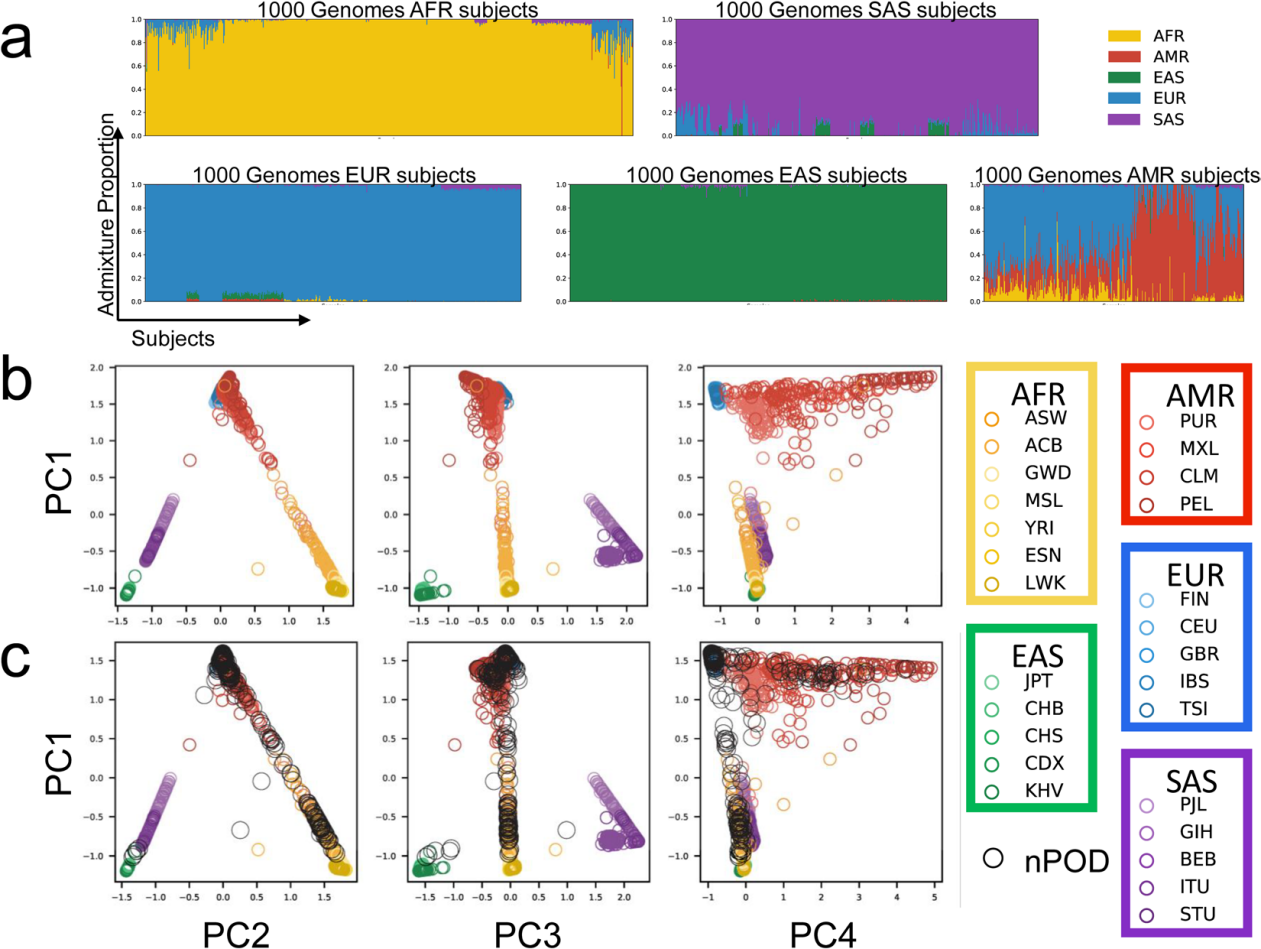
Diverse genetic ancestries of nPOD donors. (**a**) An unsupervised ADMIXTURE model for five population classes was built with the 1000 Genomes phase 3 cohort^32^ using linkage disequilibrium-pruned SNPs that overlap with the UFDIchip. Stacked bar plots of the proportion of the ancestry group assigned to each 1000 Genomes donor are shown grouped by major continental populations [African (AFR): yellow; Admixed American (AMR): red; East Asian (EAS): green; European (EUR): blue; South Asian (SAS): violet]. Four genetic ancestry classes were assigned to 1000 Genomes continental populations based on their high proportion in those groups (AFR, EAS, EUR, SAS). The fifth class was almost entirely found in the Admixed American group and was thus assigned to AMR. (**b**) While all five continental populations occupy distinct principal component (PC) space, a portion of AMR subjects are in proximity to EUR, suggesting shared genetic ancestry. (**c**) All 372 nPOD donors (black) were projected onto the 1000 Genomes reference. Americans of AFR Ancestry in SW USA (ASW, n = 112); African Caribbeans in Barbados (ACB, n = 123); Gambian in Western Divisions in the Gambia (GWD, n = 180); Mende in Sierra Leone (MSL, n = 128); Yoruba in Ibadan (YRI, n = 186); Esan in Nigeria (ESN, n = 173), Luhya in Webuye, Kenya (LWK, n = 116); Puerto Ricans from Puerto Rico (PUR, n = 115); Mexican Ancestry from Los Angeles, USA (MXL, n = 107); Colombians from Medellin, Colombia (CLM, n = 115); Peruvians from Lima, Peru (PEL, n = 130); Japanese in Tokyo, Japan (JPT, n = 105); Han Chinese in Beijing, China (CHB, n = 108); Southern Han Chinese (CHS, n = 171); Chinese Dai in Xishuangbanna, China (CDX, n = 109); Kinh in Ho Chi Minh City, Vietnam (KHV, n = 124); Finnish in Finland (FIN, n = 105); Utah Residents (CEPH) with Northern and Western European Ancestry (CEU, n = 183); British in England and Scotland (GBR, n = 107); Iberian Population in Spain (IBS, n = 162); Toscani in Italia (TSI, n = 112), Punjabi from Lahore, Pakistan (PJL, n = 158); Gujarati Indian from Houston, Texas (GIH, n = 113); Bengali from Bangladesh (BEB, n = 114); Indian Telugu from the UK (ITU, n = 118); Sri Lankan Tamil from the UK (STU, n = 128). Figure adapted from Kaddis, *et al*.^53^.

To further validate the UFDIchip data, we used the 1000 Genomes phase 3 cohort^32^ to build a reference model for genetic ancestry using ADMIXTURE software^18^ (Fig. 4a,b), projected all 372 nPOD donors onto this model to impute ancestry (Fig. 4c), and compared those results with reported race. Using methods modified from Kaddis, *et al*.^53^, we plotted PCA results of ancestry proportions and observed that each of the five major continental populations in the 1000 Genomes cohort (AFR, AMR, EAS, EUR, and SAS)^32^ clustered to occupy distinct PC space (Fig. 4b). This suggested that the five ancestry populations computed by ADMIXTURE were representative of the five continental populations from 1000 Genomes^32^. The ancestry proportions of 1000 Genomes^32^ continental populations were almost entirely represented by a single ancestry group, with the exception of admixed populations including Admixed Americans (AMR), as well as the subcontinental populations, Americans of African ancestry in SW USA (ASW) and African Caribbeans in Barbados (ACB, Fig. 4a,b), as previously observed^32^. Next, the nPOD cohort was projected onto the 1000 Genomes reference, revealing overlap with AFR, AMR, EAS, and EUR groups in PC space (Fig. 4c). Donors were then assessed for agreement between reported race and genetic ancestry, showing that the highest AFR, AMR, EAS, and EUR ancestry proportions were observed in donors reported as Black/African American, Hispanic/Latinx, Asian, and White/Caucasian respectively (**UFDIchip_admixture**.**xlsx**^27^), which is consistent with other U.S.-based admixture studies^54,55^. Notably, racial identity is complex and the method of estimating proportions of continental genetic ancestries may not adequately reflect genetic diversity^56^. With this limitation in mind, these analyses accomplish the aims of: 1) ADMIXTURE model validation using UFDIchip array data and 2) qualification of the genetic ancestry results as an alternative or additional covariate to reported race for users of this resource (**UFDIchip_admixture**.**xlsx**^27^)^57^.

### HLA imputation accuracy and concordance

The nPOD cohort was HLA typed using next generation sequencing (NGS) at *HLA-A*, *HLA-DRB1*, *HLA*-*DQA1*, and *HLA*-*DQB1*^8^ to identify donors with genotypes that are associated with T1D risk or protection^34–36^. This enables an extra level of QC and validation of the UFDIchip array data by comparing typed to genetically imputed HLA genotypes. Imputation accuracy at each locus, *Acc(L)*, was calculated assuming typed results were correct if discordant with imputed results. Overall, *Acc(L)* was >0.93 for low-resolution HLA (2-digit) and >0.90 for high-resolution HLA (4-digit) for the four loci tested (**UFDIchip_HLA_imputation_accuracy**.**xlsx**^27^).

Next, we assessed concordance between typed and imputed HLA for T1D risk (A2, A24, DR3, DQ2, DR4, DQ8, DR8, and DQ4) or protective (DR15, DQ6, and DQ7) genotypes^34–36^ (Table 3). At 2-digit resolution, all tested loci were greater than 93% concordant (median [IQR]: 98.5% [97.4%–99.8%], Table 3). At 4-digit resolution, HLA concordance was predictably lower (median [IQR]: 97.1% [92.5%–99.3%]), with notable discordance in the less common HLA-DRB1*04:xx genotypes (Table 3). Importantly, 4-digit genotypes that convert 2-digit risk to protective genotypes, such as HLA-DRB1*04:03 and HLA-DQB1*03:01, were accurately imputed with greater than 97.9% concordance (Table 3).

**Table 3:** Imputed HLA concordance with typed HLA for T1D-associated genotypes.

T1D-Associated Genotype^34–36^	T1D effect	Locus	NGS-derived 2-Digit HLA	NGS-derived 4-Digit HLA	n^#^	Concordance with Imputed HLA^#^
A2	Risk	*A*	02		200	0.995
				0201	169	0.982
A24	Risk	*A*	24		60	0.983
				2402	57	0.965
DR3	Risk	*DRB1*	03		136	0.978
				0301	128	0.992
DQ2	Risk	*DQA1*	05		204	0.985
				0501	191	0.969
		*DQB1*	02		198	0.995
				0201	147	0.898
DR4	Risk	*DRB1*	04		171	0.971
				0401	96	0.917
				0402	6	0.833
				0403^†^	1	1.000
				0404	32	0.781
				0405	13	0.923
				0407	18	0.667
DQ8	Risk	*DQA1*	03		192	0.969
				0301	183	0.973
		*DQB1*	03		265	0.996
				0302	139	0.993
DR8	Risk	*DRB1*	08		23	1.000
				0801	11	1.000
DQ4	Risk	*DQA1*	04		29	0.931
				0401	29	0.931
		*DQB1*	04		31	0.935
				0402	31	0.935
DR15	Protective	*DRB1*	15		83	1.000
				1501	60	1.000
				1503	17	0.941
DQ6	Protective	*DQA1*	01		235	0.983
				0102	117	0.983
		*DQB1*	06		132	1.000
				0602	77	1.000
DQ7^‡^	Protective	*DQA1*	03		192	0.969
				0301	183	0.973
		*DQB1*	03		265	0.996
				0301^‡^	97	0.979

For the 372 nPOD donors evaluated, the number of alleles (n) and concordance rate are displayed for donors carrying specified genotypes associated with risk or protection from T1D, as determined by high-resolution four-digit HLA typing by next generation sequencing (NGS)^8,12^.

^†^DRB1*04:03-DQ8 is protective; ^‡^DQ7 (DQA1*03:01-DQB1*03:01) is protective in DR4 haplotypes; ^#^Calculations assume that typed HLA is accurate over imputed HLA.

**Fig. 5:**
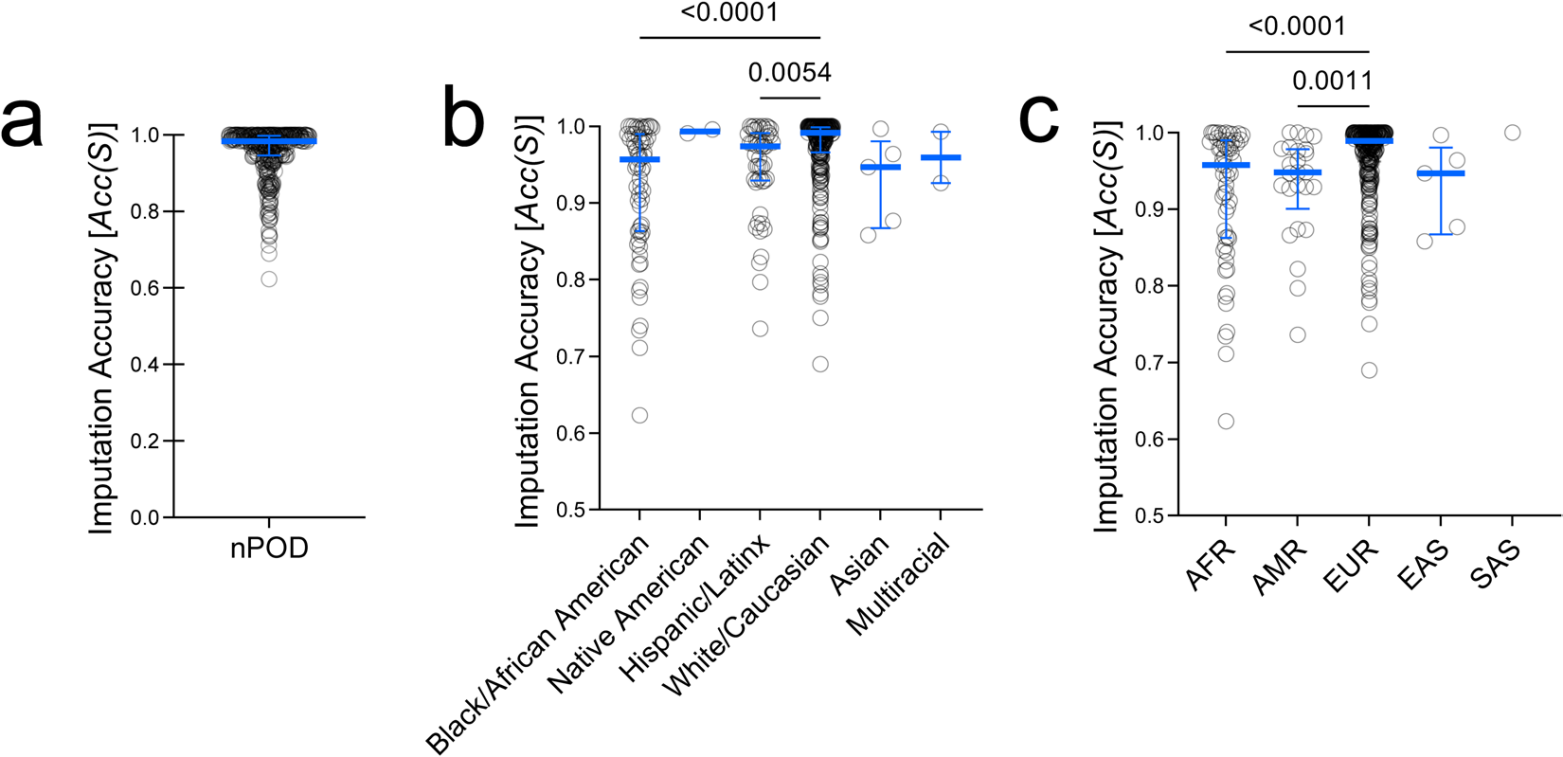
HLA imputation accuracy is decreased in non-European donors. Donor-level HLA imputation accuracy [*Acc(S)*] was calculated for *HLA-A*, *HLA-DRB1*, *HLA-DQA1*, and *HLA-DQB1* loci by comparing to next generation sequencing (NGS)-based HLA typing. *Acc(S)* is shown for (**a**) all 372 nPOD donors, (**b**) for donors grouped by reported race, and (**d**) for donors grouped by highest proportion of genetic ancestry. African (AFR), Admixed American (AMR), East Asian (EAS), European (EUR), South Asian (SAS). Bars represent median and interquartile range (IQR). Kruskal-Wallis test with Dunn’s multiple comparisons test.

Data validation at the sample level was assessed using a sample imputation accuracy score, *Acc(S)*, for 2-digit HLA at the four typed loci. *Acc(S)* was 0.984 [0.946–0.998] (median [IQR]), indicating high performance of the imputation methodology per sample (Fig. 5a). HLA imputation may be inaccurate when rare HLA genotypes and ancestrally diverse populations are underrepresented in the reference cohort^33,58,59^. In agreement with this notion, a breakdown of nPOD donors by reported race or by top genetic ancestry proportion suggests that imputation accuracy could potentially be improved with greater reference cohort diversity (Fig. 5b,c). Donors with reported race of White had significantly higher HLA imputation accuracy than those reported as Black or Hispanic/Latinx (Fig. 5b). Similarly, donors whose highest genetic ancestry proportion were EUR had higher imputation accuracy than donors whose were AFR or AMR (Fig. 5c). Notably, 4-digit HLA imputation showed 100% concordance for the 24 nPOD subjects run in technical replicate on the UFDIchip.

### T1D polygenic GRS performance using UFDIchip data

**Fig. 6:**
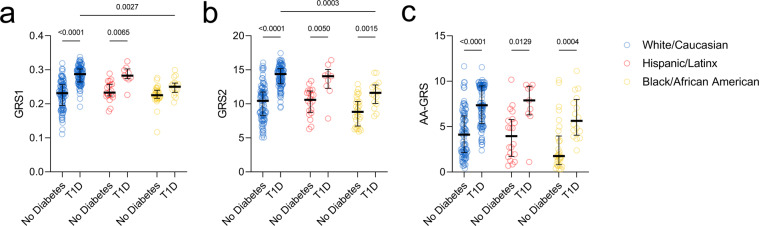
GRS2 and AA-GRS show improved performance over GRS1 at distinguishing T1D from no diabetes in non-White donors. (**a**) GRS1, (**b**) GRS2, and (**c**) AA-GRS of all 372 nPOD donors grouped by self-reported White/Caucasian (blue), Hispanic/Latinx (red), and Black/African American (yellow) no diabetes versus T1D donors. Bars represent median and interquartile range (IQR). Two-way ANOVA with Sidak’s multiple comparisons test.

Polygenic risk scores summarize genetic risk for T1D as a continuous value by aggregating estimated risk at HLA and non-HLA loci^21,22,37,38^. The original reports of GRS1 described its utility for discerning T1D from other forms of diabetes including T2D^21,60^ and MODY^22^. We previously observed that a similar GRS robustly discriminated living controls from T1D subjects reported as White but was less effective for non-White subjects, highlighting a need for diversity in risk modeling^20,53^. Shortly thereafter, GRS2 was developed to incorporate the impact of interactions between HLA haplotypes on T1D risk, showing improved discrimination of European ancestry T1D from control subjects^37^. Additionally, an AA-GRS was created to account for ancestry-specific T1D risk loci, with enhanced performance at distinguishing T1D from control subjects in AFR populations^38^. We therefore attempted to validate these previous findings regarding the ability of GRS1, GRS2, and AA-GRS to differentiate controls from T1D subjects by using the 372 nPOD cases subjected to genotyping. Indeed, White T1D donors (0.287 [0.264–0.303], median [IQR]) had significantly higher GRS1 values than White No Diabetes donors (0.231 [0.195–0.256], Fig. 6a). While Hispanic/Latinx T1D donors (0.283 [0.274–0.303]) also showed significantly higher GRS1 values than Hispanic/Latinx No Diabetes donors (0.233 [0.223–0.257]), Black T1D and No Diabetes donors were unable to be distinguished by GRS1 due to low scores in T1D donors (0.250 [0.234–0.261], Fig. 6a). In contrast, GRS2 values were significantly higher in Black T1D donors (11.62 [10.05–12.78]) than Black No Diabetes donors (8.83 [6.75–10.36]), although Black T1D donor GRS2 values remained significantly lower than those of White T1D donors (14.38 [12.94–15.16], Fig. 6b). While the AA-GRS likewise succeeded at differentiating Black T1D donors (5.634 [4.061–8.001]) from Black No Diabetes donors (1.751 [0.804–3.964]), no significant differences remained between Black T1D and White T1D donors (Fig. 6c). Taken together, these results indicate that the nPOD cohort UFDIchip array data represent a validated resource for genetic studies of T1D. Additionally, we provide GRS1, GRS2, and AA-GRS genotypes and calculated scores to the community for future studies (**GRS1_GRS2_AAGRS_TOPMed_Imputed**.**xlsx**^27^). Note that these scores differ from those provided in Kaddis, *et al*.^53^, due to updating the reference cohort for imputation from the Haplotype Reference Consortium (HRC)^61^ cohort, with predominantly European ancestry, to the TOPMed^31^ reference, with diverse genetic ancestry.

### WES

207 nPOD donors were also queried for rare variants using WES and associated data are accessible on dbGaP (see Data Records). Standard QC measures were performed to minimize adapter contamination, low-quality reads, error rate, and sequencing bias. To further validate data quality, we measured concordance between genotype calls from the UFDIchip and WES (N = 167 donors). Indeed, 27,852 autosomal biallelic variants with at least one minor allele count (MAC) in the WES data showed a concordance of 98.8% [92.2%–99.7%] (median [IQR]) with UFDIchip calls.

**Fig. 7:**
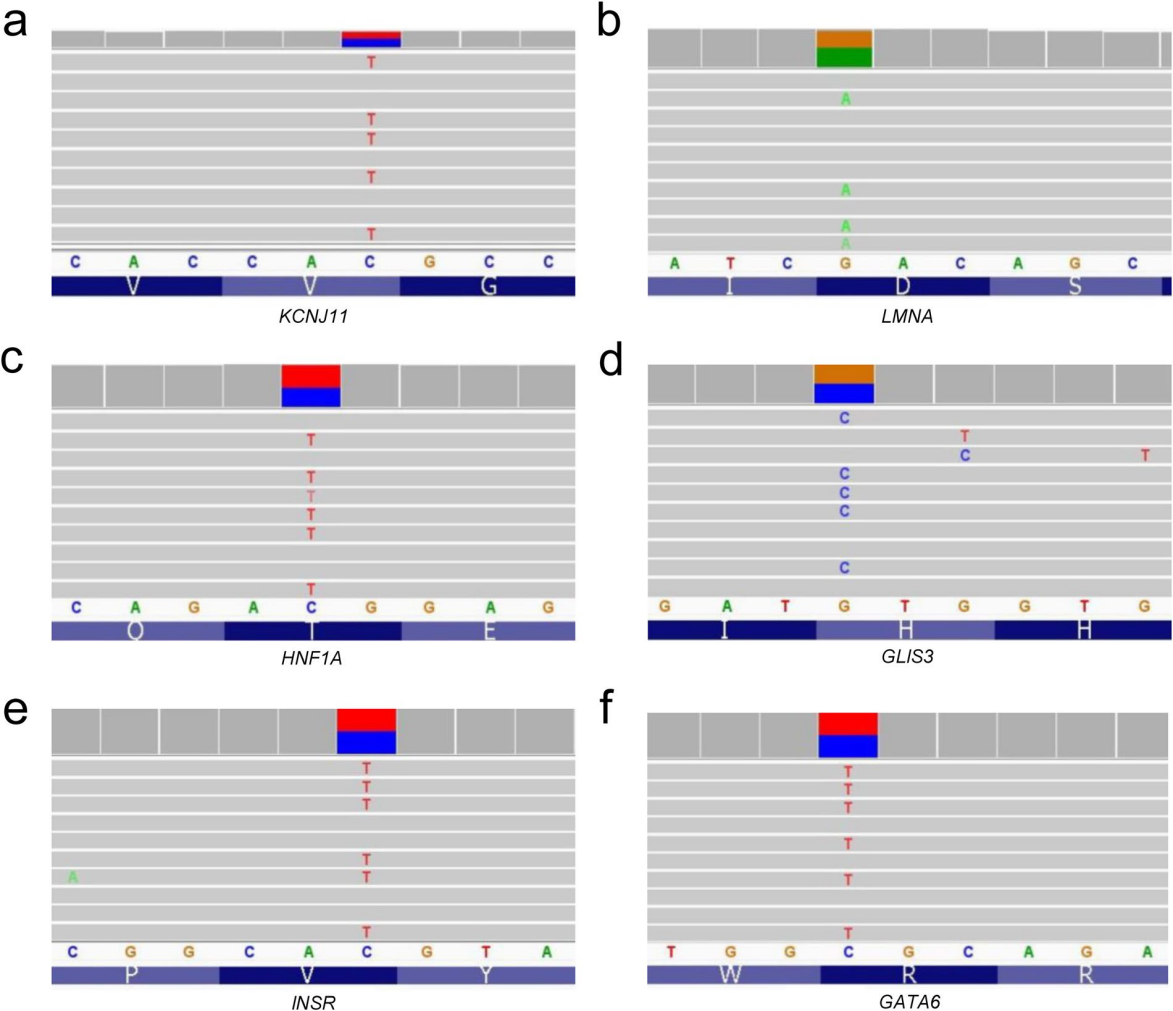
WES data identify previously published monogenic diabetes-associated gene variants in nPOD donors. Six nPOD donors were previously identified to have variants in monogenic diabetes-associated genes^67^: (**a**) *KCNJ11*, (**b**) *LMNA*, (**c**) *HNF1A*, (**d**) *GLIS3*, (**e**) *INSR*, and (**f**) *GATA6*. Alignment data viewed using the Integrative Genomics Viewer (IGV) software^63^. See Table 4 for specific gene variant information. Note that the WES reads are sometimes of the complement strand depending on gene orientation.

**Fig. 8:**
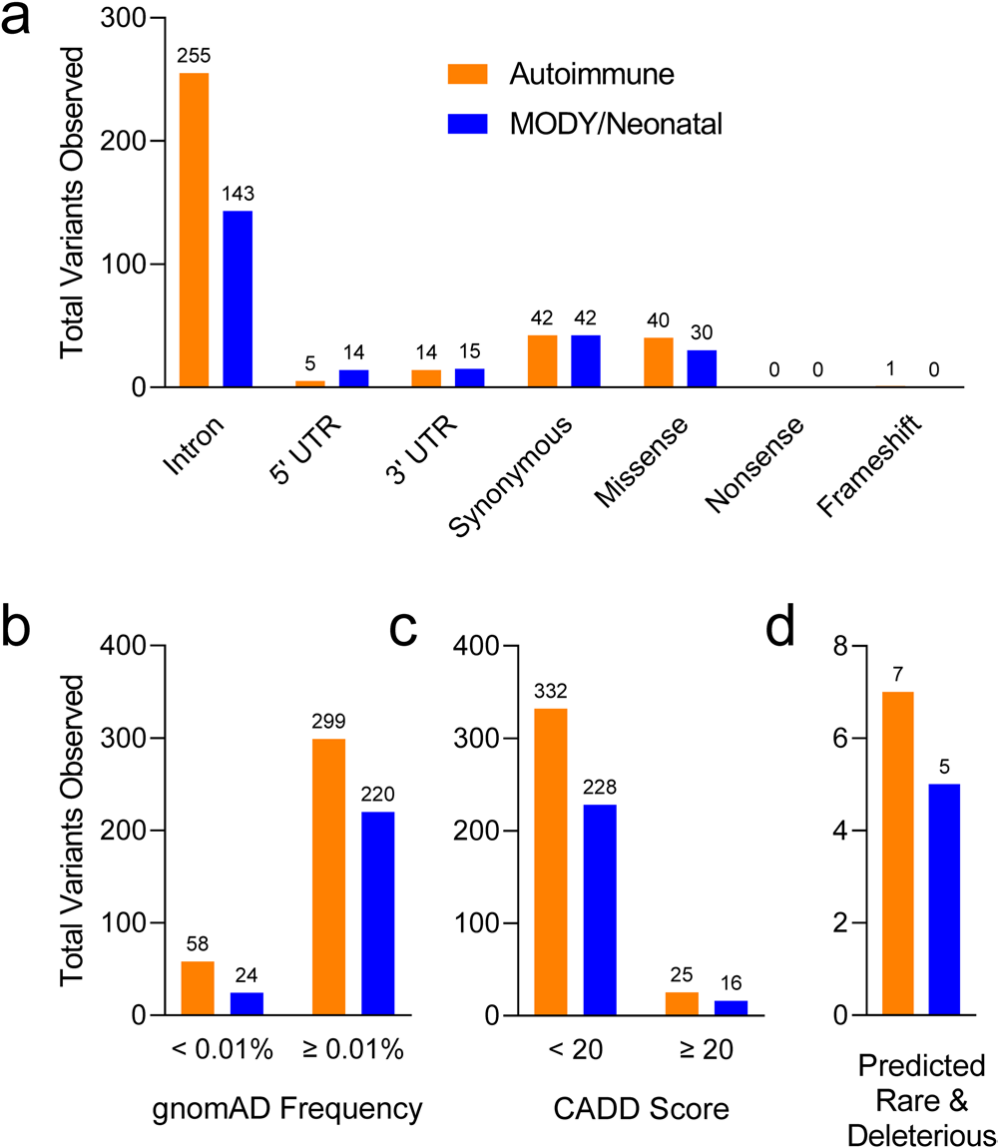
Variants in genes associated with monogenic diabetes observed in nPOD donors with T1D. Variants in genes associated with autoimmune diabetes (orange: *AIRE*, *FOXP3*, *IL2RA*, *ITCH*, *LRBA*, *SKAP2*, *STAT1*, and *STAT3*), or MODY and neonatal diabetes (blue: *GCK*, *HNF1A*, *HNF4A*, *HNF1B*, *ABCC8*, *KCNJ11*, and *INS*)^39,64–66^ were observed from WES data of 141 nPOD cases with T1D. (**a**) Distribution of variant types. (**b**) Frequency of variants in the Genome Aggregation Database (gnomAD)^47^. (**c**) Variants with a Combined Annotation Dependent Depletion (CADD) score > 20^46^, predicting the variant is deleterious to protein function. (**d**) Combining these tools can help identify variants that are predicted to be both rare (<0.01%) and deleterious. Total number of variants shown above bars.

Six of the nPOD donors were previously reported to have genetic variants with possible clinical impact in *KCNJ11*, *LMNA*, *HNF1A*, *GLIS3, INSR, and GATA6* using a custom-designed NGS panel that included 140 genes implicated in monogenic diabetes^62^. These genetic variants were validated with WES (Table 4) and visual exploration of the data using the Integrative Genomics Viewer (IGV)^63^ confirmed reads for each variant (Fig. 7). WES captures genomic DNA sequence in exons and the intronic sequence adjacent to exons. This enables the discovery of variants that directly alter the protein coding portion of mRNA (missense, nonsense, insertion/deletions) and also some regulatory intronic sequences, such as splice sites. Variants in genes associated with autoimmune diabetes (*AIRE*, *FOXP3*, *IL2RA*, *ITCH*, *LRBA*, *SKAP2*, *STAT1*, and *STAT3*) or MODY and neonatal diabetes (*GCK*, *HNF1A*, *HNF4A*, *HNF1B*, *ABCC8*, *KCNJ11*, and *INS*)^39,64–66^ were observed in 141 nPOD cases with T1D (Fig. 8a). Monogenic forms of diabetes are rare, and the vast majority of the detected variants are not expected to have functional or clinical consequences.

**Table 4:** Gene variants previously published.

Case ID	Gene	DNA Variant	Protein Variant	Zygosity	gnomAD Frequency	CADD Score
6033	KCNJ11	c.868G > A	Val209Met	Het	0.002%	23.5
6166	LMNA	c.898G > A	Asp300Asn	Het	0%	28.8
6176	HNF1A	c.29C > T	Thr10Met	Het	0.002%	22.2
6243	GLIS3	c.1863C > G	His621Gln	Het	0.001%	20.4
6264	INSR	c.3034G > A	Val1012Met	Het	0.80%	26.1
6320	GATA6	c.1366C > T	Arg456Cys	Het	0%	31

Six nPOD donors were previously identified to have variants in monogenic diabetes-associated genes^67^. See Fig. 7. gnomAD: Genome Aggregation Database, CADD: Combined Annotation Dependent Depletion.

**Fig. 9:**
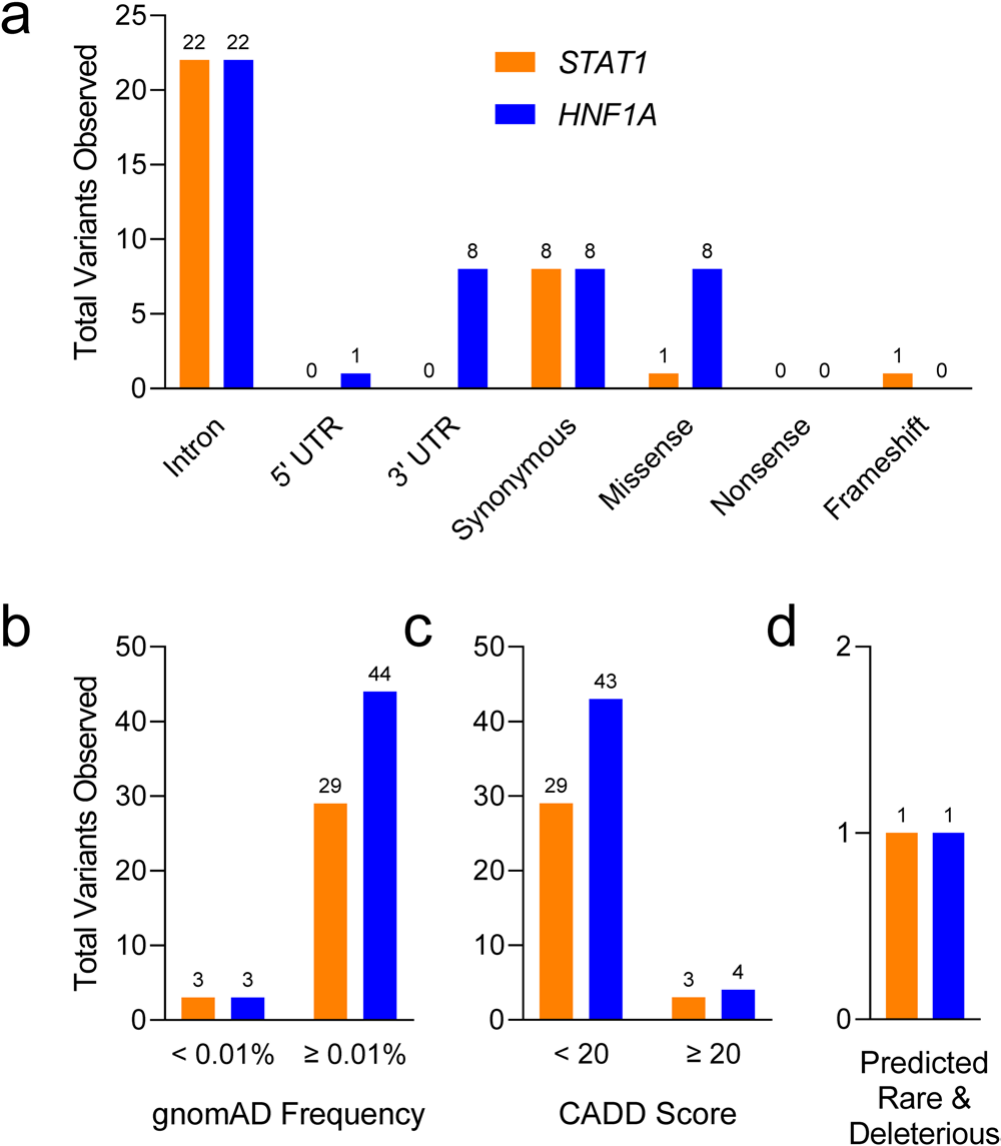
*HNF1A* and *STAT1* gene variants observed in nPOD donors with T1D. *STAT1* (orange, Autoimmune diabetes gene) and *HNF1A* (blue, MODY/Neonatal diabetes gene)^39,64–66^ variants observed from WES data of 141 nPOD cases with T1D. (**a**) Variant types, (**b**) frequency distribution of variants, (**c**) variants with CADD score ≥ 20, and (**d**) variants with CADD score ≥ 20 and gnomAD frequency < 0.01%. Note that there are two potential monogenic diabetes variants in (**d**), one in *HNF1A* and one in *STAT1*. Total number of variants shown above bars.

**Fig. 10:**
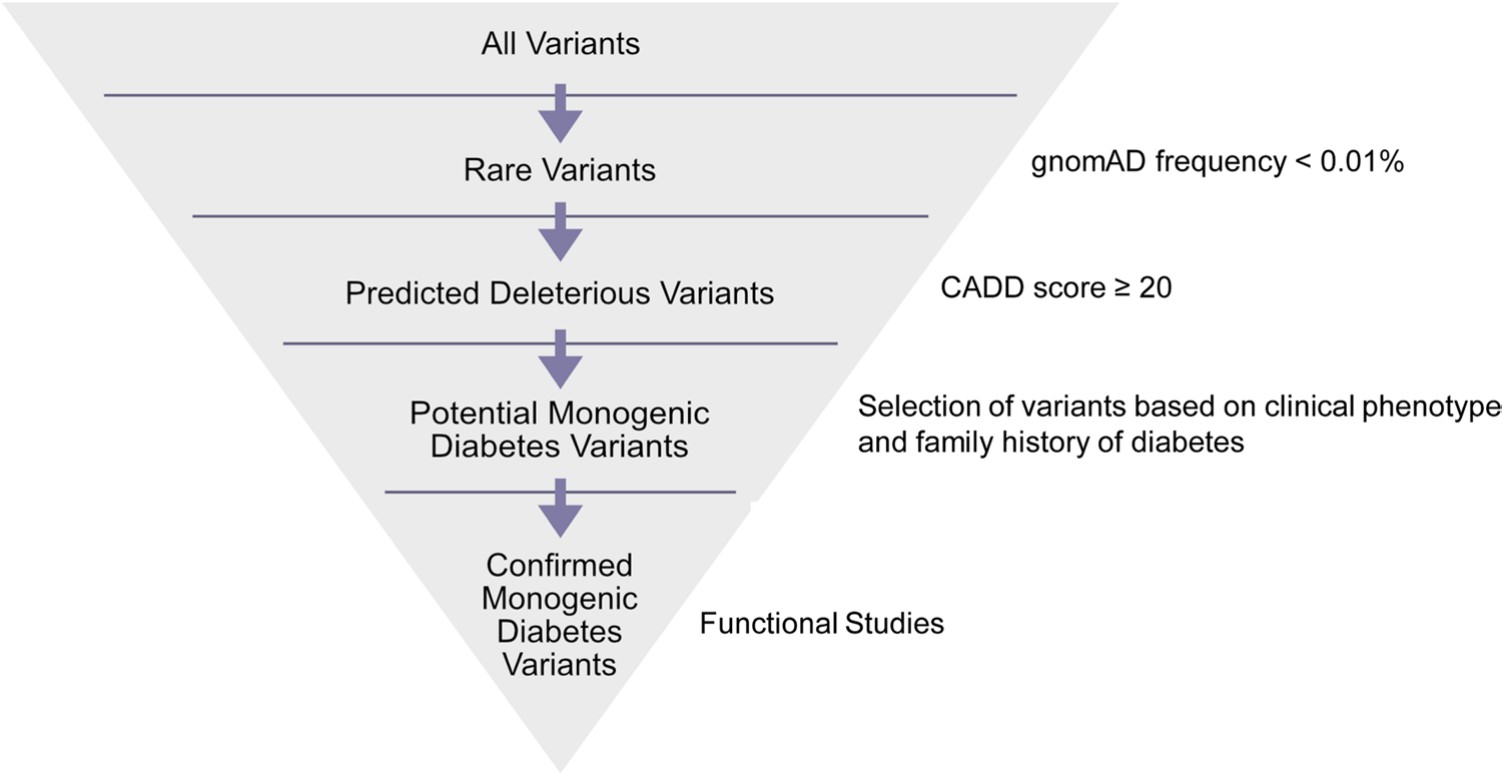
Suggested workflow for evaluating variants in monogenic diabetes genes observed in WES data. Note that functional studies are needed for potential monogenic diabetes variants that have not already been previously validated.

There are several databases and tools available to help with the identification and interpretation of genetic variants. For example, the frequency of a variant in the general population can be estimated using the gnomAD, which contains data from 140,000 + exomes and genomes from unique, unrelated individuals spanning six global ancestries^47^. Additionally, the CADD score can be used to predict severity of impact of the variants based on a variety of criteria such as sequence context, evolutionary constraint, and functional predictions^46^. As expected, the variants observed in T1D cases were distributed across a spectrum of functional classes, with only a few predicted to be both rare (frequency < 0.01%) and deleterious (CADD score ≥ 20, Fig. 8b–d). As an example of how these tools can be used, variants in the monogenic diabetes genes *HNF1A* and *STAT1* were analysed in the nPOD donors classified as T1D. One variant for each gene was predicted to be rare and deleterious based on the thresholds set for the gnomAD frequency and CADD score (Fig. 9, Table 5). The thresholds set for these and other bioinformatic tools are determined by each investigator, and are often informed by the clinical phenotype of the patient and previous knowledge about the gene’s disease association. Other variant annotations from tools including ACMG Classification^43^, SIFT Function Prediction^44^, PolyPhen-2 Function Prediction^45^, HGMD^48^, ClinVar^49^, and CentoMD^50^ are available for all 207 nPOD donors on dbGaP (see Data Records). A suggested workflow for evaluating genetic variants for potential clinical significance is shown in Fig. 10. Importantly, while computational tools facilitate interpretation, confidence in the functional or clinical relevance of the genetic variants reported herein requires rigorous experimentation.

**Table 5:** Potential monogenic diabetes variants.

Case ID	Gene	DNA Variant	Protein Variant	Zygosity	gnomAD Frequency	CADD Score
6205	*HNF1A*	c.142 G > A	p.E48K	Het	0.009%	22.1
6261	*STAT1*	c.77_80dupACAG	p.S27fs*26	Het	0.003%	24.9

Monogenic diabetes gene variants predicted to be rare and deleterious. See Figs. 8–9. gnomAD: Genome Aggregation Database, CADD: Combined Annotation Dependent Depletion.

## Usage Notes

The associated data are openly available with unrestricted access.

## Data Availability

No custom code or scripts were used for the curation and validation of the dataset.
